# Supplementary material for: Commuting network effect on urban wealth scaling
Source: Sci Rep. 2021 Nov 25;11:22918. doi: 10.1038/s41598-021-02327-7 (PMC8617032; doi:10.1038/s41598-021-02327-7)
Supplement: Supplementary file 1 — Supplementary Figure S1. [file 41598_2021_2327_MOESM1_ESM.pdf]

Supplementary Information for

Commuting network effect on urban wealth scaling

Luiz G. A. Alves, Diego Rybski, and Haroldo V. Ribeiro

Scientific Reports, 2021

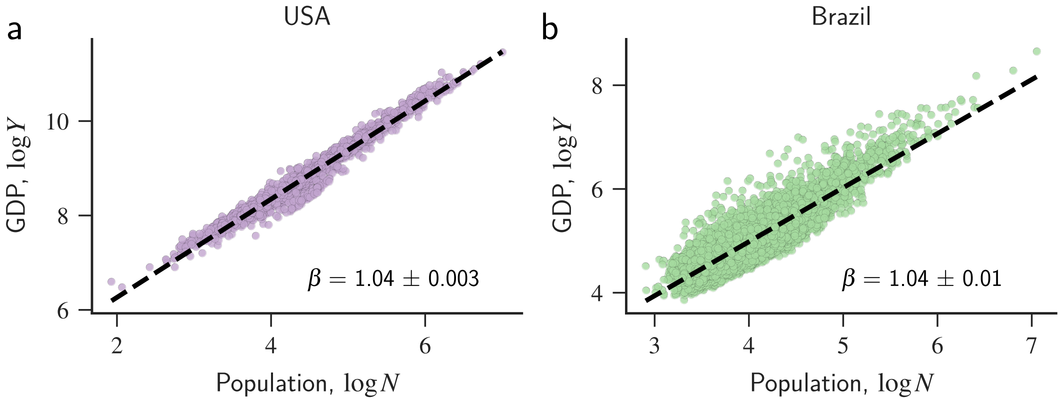

Figure S1. Scaling relationships between urban GDP (Y) and population size (N) for US counties (purple markers of panel a) and Brazilian municipalities (green markers of panel b). The dashes lines represent power-law fits (Eq. 1) with exponents β indicated within each panel.
